# Evaluation of open reduction of distal humerus fractures in children after implementation of an enhanced recovery after surgery program

**DOI:** 10.1186/s12891-022-05675-1

**Published:** 2022-07-25

**Authors:** Jialing Lu, Mingfeng Xue, Peng Fu, Damei Qian, Xingguang Chen, Danhua Yao, Yanli Zhang

**Affiliations:** 1 Department of Pediatric Orthopedics, The Second Affiliated Hospital of Jiaxing University (The Second Hospital of Jiaxing), Zhejiang Jiaxing, 314000 People’s Republic of China; 2 Department of Anesthesiology, The Second Affiliated Hospital of Jiaxing University (The Second Hospital of Jiaxing), Zhejiang Jiaxing, 314000 People’s Republic of China

**Keywords:** Enhanced recovery after surgery, Distal humerus fractures

## Abstract

**Objective:**

This study assessed whether an enhanced recovery after surgery (ERAS) protocol could be beneficial for children with distal humerus fractures.

**Methods:**

Children with distal humerus fractures (*n* = 85) were randomly assigned to the ERAS and control groups and subjected to different perioperative managements. This was followed by the evaluation of their intraoperative characteristics (operation time and bleeding), postoperative characteristics (food intake conditions, pain scores, and discharge time), and postoperative functions.

**Results:**

The operation time, intraoperative bleeding, and postoperative hematological indices did not differ significantly between the two groups. Preoperative thirst and hunger were considerably less and the initial food intake duration following surgery was markedly shorter in the ERAS group than in the control group, whereas no difference between the groups was observed in the incidences of postoperative nausea and vomiting. A markedly reduced highest postoperative pain score and reduced mean pain score and demand for additional analgesic interventions were observed in the ERAS group compared with those in the control group, although the differences were not statistically significant. No noticeable between-group differences were observed in the incidences of postoperative incision problems, aspirational pneumonia, and gastroesophageal reflux. The total length of hospital stay was not significantly different between the two groups. However, the length of postoperative hospital stay was remarkably shorter and the elbow joint function at 2 months after surgery was significantly improved in the ERAS group compared with those in the control group.

**Conclusion:**

The ERAS protocol can ameliorate preoperative discomfort and postoperative pain, shorten the postoperative hospital stay, and accelerate postoperative functional recovery without increasing the risks of postoperative nausea, vomiting, and poor incision healing and is, therefore, worthy of clinical application.

Distal humerus fractures account for approximately a quarter of fractures occurring in children [1, 2] and are among the most common fractures in children. These fractures can easily lead to complications such as elbow deformity, dysplasia, and joint stiffness in children, resulting in abnormal appearance and function, which which necessitates aggressive therapeutic approaches. Most intra-articular and some extra-articular distal humerus fractures require surgical treatment, with some patients requiring open reduction. Children who undergo open reduction may experience longer hospital stays and greater risks of postoperative complications than those who undergo closed reduction [3]. This study introduces the concept of enhanced recovery after surgery (ERAS), compares the effects of different modes of perioperative management on postoperative efficacy and complications in children with distal humerus fractures, and explores the application of an ERAS protocol in the pediatric orthopedics field.

## Materials and methods

### General data

Children with distal humerus fractures who were admitted to the Pediatric Orthopedics Department of the Second Affiliated Hospital of Jiaxing University from January 2021 to January 2022 were enrolled. Patients aged < 14 years, with displaced lateral humeral condyle fractures or supracondylar humerus fractures who were scheduled to undergo surgical treatment were included in the study. Patients with open fractures or open trauma around the elbow joint, pathological fractures, dated fractures which remained more than 2 weeks, successful closed reduction during surgery, and serious underlying diseases or congenital deformities that may affect treatment, and those already on any opioid-containing medications were excluded. The enrolled children were randomly allocated to the ERAS and control groups by applying the closed envelope method. After admission, surgeons and nurses in charge of the ERAS process participated in the reception of children in the ERAS group, and the anesthetists, operating room nurses, and nutritionists participated in their diagnosis and treatment. The control group received traditional perioperative management. The management schedules of both groups are described in Table 1.

**Table 1:** ERAS and general schedules for fractures around the elbow in children

	ERAS schedules	General schedules
Admission		
Parent education	Dietary and analgesic guidance, and alleviation of parents’ anxiety	Conventional admission education
Analgesia	Oral analgesia	No requirement
Nutrition	Pediatric nutritional risk assessment (PNYS) and nutrition intervention	No requirement
Detumescence	Elevation, cold compresses, fist-clenching guidance, without decongestants	Elevation and ice compresses in the presence/absence of decongestants, decided by surgeons
Before surgery		
Skin preparation	Preoperative skin cleaning without hair removal	No requirement
Fasting and water deprivation time	No light drinking for 2 h, no breast milk for 4 h, no solid carbohydrates for 6 h, and no fat or protein intake for 8 h	Fasting and water deprivation for 8 h
Carbohydrate loading^a^	Intravenous infusion of 4:1 glucose and sodium chloride after fasting	Fluid supplementation for hungry pediatric patients
Catheterization and intestinal preparation	No catheterization and intestinal preparation	Decided by surgeons
Nerve block	Interscalene brachial plexus block	No requirement
During surgery		
Tubing and drainage	No incision drainage tube or reduction of other tubes	Drainage tube use decided by surgeons
Body temperature	Contimuous body temperature monitoring and thermostatic blanket use during surgery	No requirement of continuous monitoring
Reduce the risk of bleeding	Tourniquet use during surgery	No requirement
Analgesia	Interscalene brachial plexus block, analgesic pump, and regular oral pain medications	Pain medication as required, an analgesic pump decided by parents, no requirement for nerve block
After surgery		
Food intake	Fasting after waking	Fasting and water deprivation after waking for 2 h
Tube drawing	Drawing of infusion tube 1 day after surgery	Tube drawing time point decided by surgeons
Nutrition	Reassessment and intervention after surgery	No requirement
Carbohydrate loading	Discontinuation of parenteral nutrition 1 day after surgery	Energy supplement discontinuation time point decided by surgeons
Postoperative dressing change and discharge	Dressing change on the 2nd and 4th days and discharge on the 4th day	Decided by surgeons
	Removal of Kirschner wires and plaster as well as functional exercise guidance in the 4th–5th weeks	Removal time of fixtures decided by surgeons
Post-discharge compliance	Regular telephone and outpatient follow-up after discharge	Regular outpatient follow-up after discharge

^a^Whether to give intravenous infusion during preoperative fasting

### Surgical methods

**Fig. 1:**
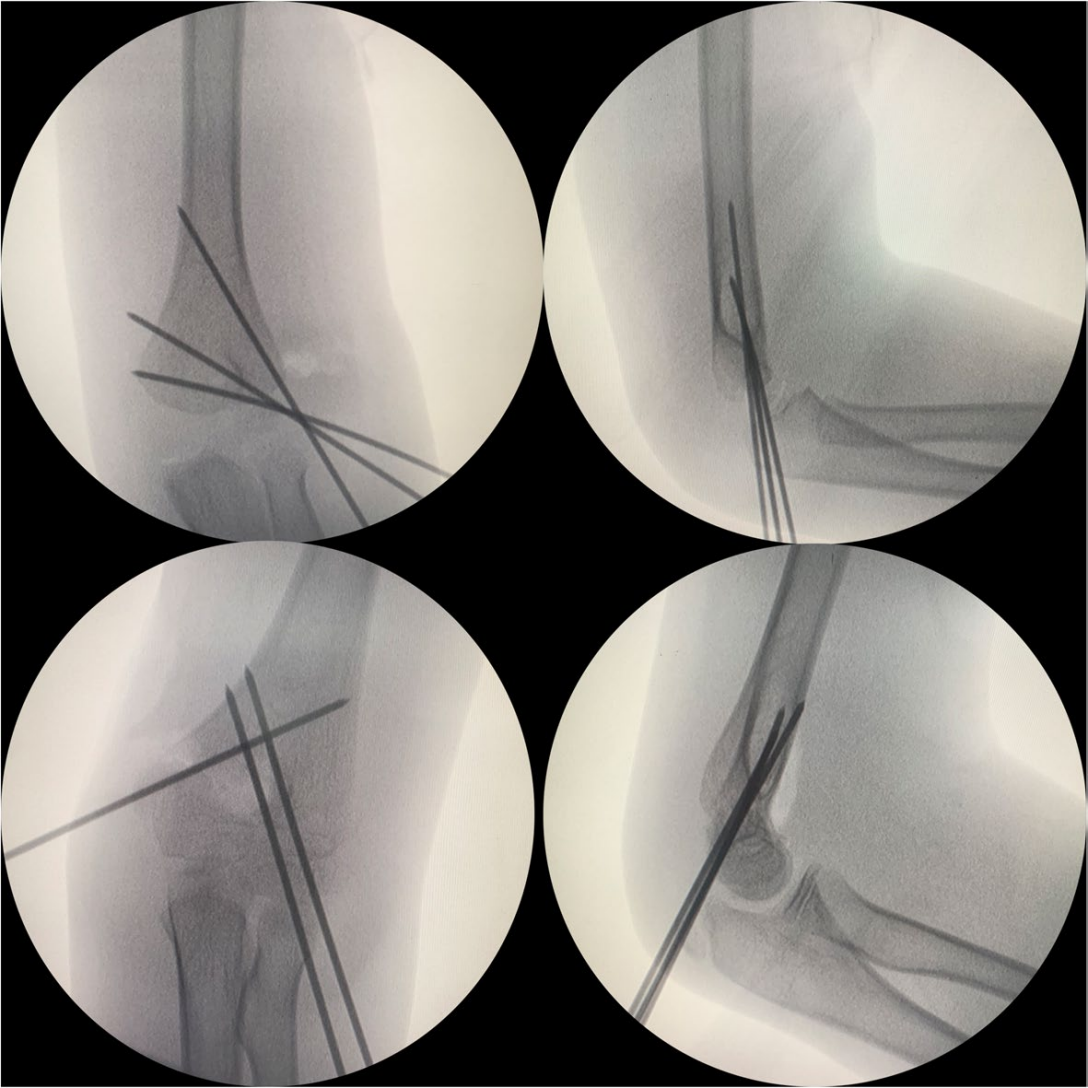
Two internal fixation approaches for distal humerus fractures

Surgery was performed by the same medical team. A medial incision of the elbow joint was employed for supracondylar humerus fractures, and a lateral incision of the elbow joint was applied for lateral humeral condyle fractures. Both traction and poking manipulations were used for reduction. During the surgery, percutaneous fixation was performed with titanium Kirschner wires (diameter: 1.5 mm). For the supracondylar humerus fractures, two Kirschner wires were inserted from the lateral condyle and one wire was inserted from the medial epicondyle, crossed through the fracture end, and fixed in the contralateral cortex. For the lateral humeral condyle fractures, three Kirschner wires were inserted from the lateral condyle and fixed radially through the fracture end to the contralateral cortex (Fig. 1). During the surgery, a C-arm X-ray machine was used to verify fracture reduction and fixation, and the incision was sutured using absorbable sutures layer by layer. Polymer plaster was used for fixation from the shoulder to the metacarpophalangeal joint, and the elbow joint was fixed at 90°.

### Outcome measures and assessment tools

Preoperative characteristics (general characteristics, affected limb, fracture classification, hematological indices, nutritional status, and discomfort), intraoperative characteristics (operation time and bleeding), postoperative characteristics (food intake, glucose level, pain score, complications, discharge time, and requirement of additional painkillers for pain relief), opiods usage (all switched into equivalent dose of morphine), follow-up data, and compliance data of the children were collected. The PNYS scale was used for nutritional risk assessment. The fractures were classified using the Gartland and Jakob classification methods. Pain was evaluated using the Face, Legs, Activity, Cry, and Consolability scale in patients under 7-yaer-old and NRS score in those above 7-year-old. The modified Mayo elbow performance score was used for assessing elbow function.

### Statistical methods

Statistical analysis was performed using IBM SPSS Statistics version 23.0 software. The *t* test was employed for analyzing the measurement data, and the chi-square test was used for analyzing the enumeration data. A *p* value of < 0.05 was considered statistically significant.

## Results

In total, 154 children with distal humerus fractures, including 10 with supracondylar humerus fractures and 53 with lateral humeral condyle fractures, were recruited in this study. Of the total, 69 pediatric patients with successful closed reduction, including 67 with supracondylar humerus fractures and 2 with lateral humeral condyle fractures, were excluded. Finally, 85 children were selected for the study, and no statistically significant difference was noted in their baseline characteristics and fracture classification (Tables 2 and 3).

**Table 2:** Baseline characteristics of pediatric patients in the two groups

	ERAS group	Control group	*P*
Age (years)	6.6 ± 3.7	6.2 ± 3.1	0.554
Gender (n)			0.637
Boys	15	16	
Girls	29	25	
Affect limbs (n)			0.770
Left	19	19	
Right	25	22	
Diagnosis (n)			0.535
Supracondylar humerus fractures	19	15	
Lateral humeral condyle fractures	25	26	
Hematological indices			
Hemoglobin(g/L)	120.8 ± 11.1	120.6 ± 11.6	0.932
Albumin(g/L)	42.2 ± 2.2	43.1 ± 2.4	0.110
Prealbumin(mg/L)	172.1 ± 30.6	168.7 ± 41.0	0.637
Nutrition assessment (n)			
Low risk	44	39	
Medium-to-high risk	0	2	0.443

**Table 3:** Fracture classification of pediatric patients in the two groups

	ERAS group	Control group	*P*
Supracondylar humerus fractures (n)			
III	19	15	-
Lateral humeral condyle fractures (n)			
II	6	9	
III	19	17	0.406

Regarding preoperative discomfort, the ERAS group experienced less thirst and hunger than the control group. No significant differences in operation time, intraoperative bleeding, and postoperative hematological indices were observed between the two groups. The initial food intake duration after surgery was remarkably shorter in the ERAS group than in the control group, with no significant difference in the incidences of postoperative nausea and vomiting (PONV) between the groups. For pain control, the application of patient-controlled analgesia (PCA) notably increased in the ERAS group compared with that in the control group. The highest postoperative pain score was markedly reduced in the ERAS group compared with that in the control group. Although the mean pain score and the need for additional analgesic interventions were reduced in the ERAS group compared with those in the control group, the differences were not statistically significant. Laboratory examination revealed no significant difference in hemoglobin, white blood cells, CRP, PCT, albumin, arealbumin and glucose level between the two groups. Following surgical treatment, 2 patients in the ERAS group and 3 patients in the control group developed persistent incision exudation, with a second surgery required for 1 case of fat liquefaction in the control group. No significant difference in the total opiods usage between 2 groups was observed, but the ERAS group showed less opoid usage per kilogram of body weight than the control group. No patient exhibited aspirational pneumonia, and 3 patients developed gastroesophageal reflux (2 in the ERAS group and 1 in the control group, *p* = 0.599). The postoperative hospital stay was shorter in the ERAS group than in the control group; however, the difference was not statistically significant (Table 4).

**Table 4:** Intraoperative and postoperative characteristics of pediatric patients in the two groups

	ERAS group	Control group	*P*
Preoperative discomfort			
Dizziness (n)	6	9	0.315
Gastrointestinal discomfort (n)	3	5	0.634
Thirst (n)	3	13	0.003
Hunger (n)	17	33	< 0.001
Operation situation			
Operation time (h)	1.2 ± 0.6	1.2 ± 0.5	0.754
Intraoperative bleeding (mL)	7.5 ± 7.9	5.7 ± 4.2	0.185
Food intake			
Initial food intake duration after surgery (h)	2.0 ± 1.3	2.7 ± 1.8	0.041
PNOV (cases)	1	1	1.000
Pain			
PCA (cases)	32	14	< 0.001
Mean pain score	1.8 ± 0.3	2.0 ± 0.6	0.061
Highest pain score	2.0 ± 0.5	2.6 ± 1.4	0.004
Additional interventions (cases)	5	11	0.068
Opiods usage			
Total dose (mg)	74.4 ± 39.4	92.3 ± 45.4	0.057
Dose per kilogram (mg/kg)	3.00 ± 1.37	3.97 ± 2.07	0.013
Hematological indices			
Hemoglobin (g/L)	116.6 ± 20.0	113.8 ± 11.8	0.427
White blood cells (× 10^9^/L)	10.0 ± 2.6	10.6 ± 2.1	0.328
CRP (mg/L)	12.6 ± 16.9	9.9 ± 9.9	0.383
PCT (μg/L)	0.11 ± 0.12	0.08 ± 0.05	0.369
Albumin (g/L)	39.4 ± 2.7	40.4 ± 2.7	0.110
Prealbumin (mg/L)	158.9 ± 34.0	155.5 ± 29.7	0.626
Preoperative glucose (mmol/L)	4.63 ± 0.47	4.59 ± 0.40	0.694
Postoperative glucose (mmol/L)	4.65 ± 0.41	4.55 ± 0.43	0.288
Perioperative complications			
Incision problems (cases)	2	4	0.608
Aspirational pneumonia (cases)	0	0	-
Gastroesophageal reflux (cases)	2	1	0.599
Hospital stays			
Total length of hospital stay (days)	7.1 ± 2.0	6.7 ± 2.6	0.483
Length of postoperative hospital stay (days)	4.09 ± 0.88	4.95 ± 2.49	0.034

**Fig. 2:**
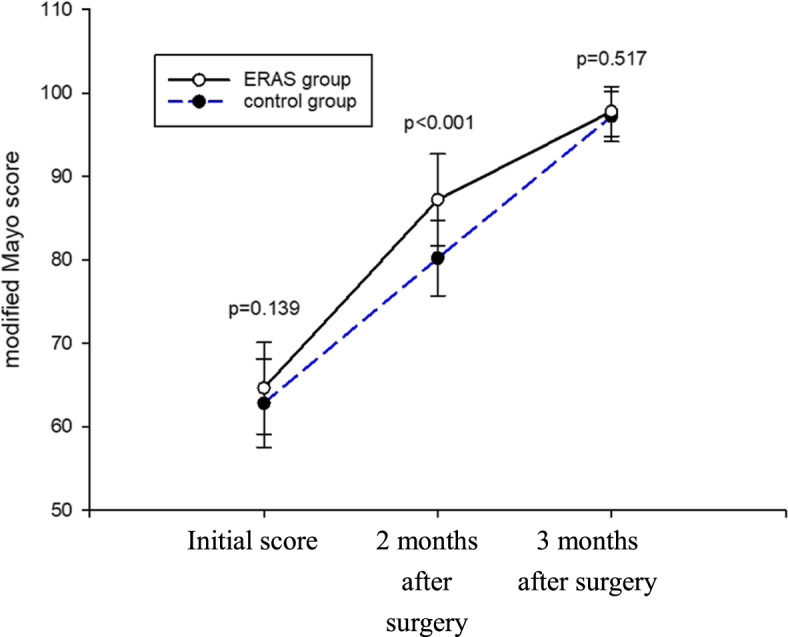
Comparison of postoperative elbow function between the two groups

No noticeable difference in the initial postoperative function score after plaster removal was observed between the two groups. Two months after the surgery, the ERAS group exhibited a markedly higher postoperative function score than the control group; however, no significant difference in the score was observed between the two groups 3 months after the surgery (Fig. 2).

## Discussion

The core concept of ERAS, which was first proposed by Henrik Kehlet in the 1990s [4], is to accelerate patient recovery by reducing physical and mental stress during the perioperative period through evidence-based treatment methods and multidisciplinary collaboration. Relatively fewer studies have investigated ERAS application in the field of pediatric surgery, mainly general surgery. A meta-analysis in 2016 included five studies pertaining to the application of the ERAS protocol in 502 children who underwent surgery; however, they mainly discussed about gastrointestinal surgery and urological surgery [5]. A Hannover Medical School team in Germany conducted several investigations on items such as hospital stay, parent satisfaction, pain management, nutrition, early mobilization, application of minimally invasive techniques, and complications [6]. They proposed that ERAS protocols should be pertinently implemented depending on the specific conditions of the pediatric patients, which can also benefit children’s accelerated rehabilitation. However, ERAS application in pediatric orthopedics remains limited. Hong Pan from Huazhong University of Science and Technology discussed ERAS protocols for supracondylar humerus fractures in children [7], yet only a few randomized controlled trials (RCTs) have used these protocols.

### Pain management

Pain, as the fifth vital sign, can directly affect the medical experience and cooperation of pediatric patients. Reducing perioperative pain is crucial for reducing stress among children and promoting their recovery. Perioperative pain management requires joint participation of the surgical department, anesthesiology department, and nurses. Pain management has been considered a noteworthy part of the ERAS process in multiple maturely applied ERAS programs, and Chinese expert consensus on anesthesia has proposed multimodal analgesia during surgery as one of the core contents of ERAS for children recovering after surgery in 2021 [8]. We, herein, adopted a multimodal analgesic regimen of preoperative brachial plexus block, postoperative PCA, and regular oral analgesics for pediatric patients in the ERAS group, aiming to reduce their pain and opioid use.The ERAS group showed less opoid usage per kilogram of body weight than the control group(*p* = 0.013). The highest postoperative pain score was lower in the ERAS group than in the control group (*p* = 0.009), and the mean pain score and the demand for additional analgesic interventions were also relatively decreased in the ERAS group compared with those in the control group (*p* = 0.069 and *p* = 0.068, respectively). Large-scale long-term studies with higher compliance may be required for further validation of this issue.

### Dietary management

Children, especially infants, have poorer tolerance to perioperative fasting and water deprivation than adults. Traditional 6–8 h of preoperative fasting and water deprivation can easily result in not only problems such as dehydration but also obvious thirst and hunger in children. A fasting time of up to 6 h for solid foods, up to 4 h for breast milk, and up to 2 h before surgery for light drinking was proposed by the European Society of Anesthesiology in 2011. As described in the 2017 American Society of Anesthesiologists guidelines, patients should be deprived of solid foods such as fat and meat for 8 h and light drinking 2 h before anesthesia [9]. The 2021 Chinese anesthesia expert consensus recommended a fasting and water deprivation regimen consisting of withholding solid food for 6 h, breast milk for 4 h, light drinking for 2 h, and oral carbohydrate-rich liquids for 2 h before surgery [8]. Based on the aforementioned viewpoints, this study used a regimen including no light drinking for 2 h, no breast milk for 4 h, no solid carbohydrates for 6 h, no fat and protein for 8 h, and no oral consumption of carbohydrate-rich liquids for 2 h. The ERAS group experienced less thirst and hunger than the control group. No intraoperative aspirational pneumonia or gastroesophageal reflux was observed in the current study.

Traditionally, anesthetics, especially opioid drugs, are considered to easily cause gastrointestinal paralysis. Therefore, postoperative fasting is also required for non-gastrointestinal surgery to allow the recovery of gastrointestinal function and prevent PONV incidences. With the development of multimodal analgesia and the decline in the use of opioid drugs, this measure faces a new challenge. Some scholars believe that children can eat when awake with a willingness to eat [10]. Early ambulation and early feeding have been recommended by the Chinese expert consensus on anesthesia [8]. In this study, the aforementioned scheme was adopted for the ERAS group as they ate immediately after waking, and we found no distinct difference in PONV incidences between the ERAS and control groups.

### Length of hospital stay

Reducing the hospitalization time of pediatric patients has always been one of the ERAS goals. Many retrospective studies and related meta-analyses at home and abroad have indicated that the ERAS protocol can strikingly shorten the length of hospital stay of pediatric patients [11, 12]; however, these studies have mostly focused on abdominal surgery, and evidence from RCTs is lacking. The present work unveiled that the ERAS program shortened the postoperative hospital stay of pediatric patients by approximately 1 day, but it did not significantly shorten the total hospital stay, likely relating to the generally mild disease, fast recovery, short hospitalization time in children with distal humerus fractures. Moreover, implementation of the ERAS protocol may increase the preoperative preparation time, making effective reduction of the total hospitalization time of children difficult.

### Tube indwelling

The ERAS concept advocates that the number of indwelling tubes and indwelling duration should be reduced. Drainage tubes and strips can easily increase fear among children, and drainage removal also increases their pain. In the current study, no incision drainage was used for the pediatric patients in the ERAS group, and consequently, no noticeable difference was observed in postoperative incision problems between the two groups. The placement of incision drainage tubes does not reduce the incidences of incision problems, such as incision exudation, fat necrosis, and infection, in children with distal humeral fractures, following open reduction and internal fixation.

In this study, the indwelling needles of all patients in the ERAS group were removed on the first postoperative day, and the common postoperative hematological indicators displayed no significant difference between the two groups. Because children with distal humerus fractures have slight gastrointestinal disturbance, early-stage feeding and reducing intravenous medication after surgery will not markedly affect the internal environment of such pediatric patients. Reduction of infusion and early removal of indwelling needles can contribute to early ambulation in pediatric patients, accelerating their recovery.

### Functional rehabilitation

Distal humerus fractures in children can easily lead to varus deformity of the elbow joint, joint stiffness, and abnormal development of the upper limbs. Hence, postoperative functional exercise is particularly vital. For children with distal humerus fractures undergoing open reduction and internal fixation with Kirschner wires, early removal of the plaster, removal of Kirschner wires, and functional exercises are beneficial for elbow joint function recovery. The plaster removal time and Kirschner wire fixation time have always been controversial topics among pediatric orthopedists. Most scholars consider that the Kirschner wire should be removed within 4–6 weeks, and some scholars use a brace for fixation after removing the plaster [13].

In this study, the plaster and Kirschner wires in the ERAS group were removed 4 weeks after surgery. After plaster removal, the elbow joint function was immediately assessed on the basis of the Mayo score, which showed moderate improvement in elbow joint function in both groups without any significant difference. At 2-month follow-up after surgery, a good elbow joint function was observed in both groups, with the ERAS group exhibiting a remarkably higher score. At 3-month follow-up following surgery, both groups exhibited an excellent elbow joint function. Thus, the ERAS protocol was suggested to accelerate elbow joint function recovery in pediatric patients, allow an earlier return to normal life, and reduce nursing dependence.

### Limitations and perspectives

Some study results were not statistically significant, which may be attributed to the limited sample size of this study, and these results should be further validated through a multicenter, large-scale study. Moreover, our study had a short follow-up period, and some patients were lost to follow-up. Therefore, the mid- and long-term efficacies of the ERAS protocol could not be evaluated. Additional long-term follow-up data are required to verify the impact of the ERAS protocol on the physical function, need for readmission, and growth and development of children after 3 months.

## Conclusion

The ERAS protocol can alleviate postoperative pain, shorten the postoperative hospital stay, and expedite the restoration of postoperative functions without increasing the risks of PONV and incision problems in children undergoing surgery for distal humerus fractures. Therefore, the ERAS protocol is valuable for clinical application.

## Data Availability

The datasets generated during and/or analyzed during the current study are available from Jialing Lu (ljl6445@126.com) on reasonable request.

## References

[1] Cheng JC, Lam TP, Shen WY (1995). Closed reduction and percutaneous pinning for type III displaced supracondylar fractures of the humerus in children. J Orthop Trauma.

[2] Hanlon CR, Estes WL (1954). Fractures in childhood, a statistical analysis. Am J Surg.

[3] Hussein Al-Algawy AA, Aliakbar AH, Witwit IHN (2019). Open versus closed reduction and K-wire fixation for displaced supracondylar fracture of the humerus in children. Eur J Orthop Surg Traumatol.

[4] Kehlet H (1997). Multimodal approach to control postoperative pathophysiology and rehabilitation. Br J Anaesth.

[5] Shinnick JK, Short HL, Heiss KF, Santore MT, Blakely ML, Raval MV (2016). Enhancing recovery in pediatric surgery: a review of the literature. J Surg Res.

[6] Reismann M, Dingemann J, Wolters M, Laupichler B, Suempelmann R, Ure BM (2009). Fast-track concepts in routine pediatric surgery: a prospective study in 436 infants and children. Langenbecks Arch Surg.

[7] Hong P, Tang X, Ruijing Xu, Li J (2019). Application and prospect of the concept of accelerated rehabilitation surgery in children’s orthopedics. J Clin Pedi Surg.

[8] Chinese Society of Cardiothoracic and vascular anesthesia, and Pediatric anesthesiology branch of Chinese Medical Association. Chinese expert consensus on anesthesia in pediatric accelerated rehabilitation surgery. Chin Med J. 2021;101(31):2425–2432.

[9] Practice guidelines for preoperative fasting and the use of pharmacologic agents to reduce the risk of pulmonary aspiration: application to healthy patients undergoing elective procedures: an updated report by the American Society of Anesthesiologists Task Force on preoperative fasting and the use of pharmacologic agents to reduce the risk of pulmonary aspiration. Anesthesiology. 2017;126(3):376–393. 10.1097/ALN.0000000000001452. 10.1097/ALN.0000000000001452 28045707

[10] Li C, Li T, Wang D, Zhang T, Xiaohua Su (2019). Practice and Exploration of standardized management of pediatric day surgery. Chin J Hospital Manag.

[11] Zhu T, Zhu D, Hongxing Yu, Feng J (2019). A meta-analysis of accelerated rehabilitation surgery in the treatment of acute appendicitis in children. Chin J Pediatr Surg.

[12] Li J, Rai S, Ze R, Tang X, Liu R, Hong P (2020). Enhanced recovery care versus traditional non-ERAS care following osteotomies in developmental dysplasia of the hip in children: a retrospective case-cohort study. BMC Musculoskelet Disord.

[13] Chen Q, Gong Y, Jianghui Gu (2014). Comparison of two surgical approaches in the treatment of complex humeral supracondylar fractures in children. Chin J Hand Surg.

